# Fine temporal structure of neural synchronization

**DOI:** 10.1186/1471-2202-14-S1-P336

**Published:** 2013-07-08

**Authors:** Sungwoo Ahn, Leonid L Rubchinsky

**Affiliations:** 1 Department of Mathematical Sciences and Center for Mathematical Biosciences, Indiana University Purdue University Indianapolis, Indianapolis, IN 46202, USA; 2 Stark Neurosciences Research Institute, Indiana University School of Medicine, Indianapolis, IN 46202, USA

## 

While neural synchronization is widely observed in neuroscience, neural oscillations are rarely in perfect synchrony and go in and out of phase in time. Since this synchrony is not perfect, the same synchrony strength may be achieved with markedly different temporal patterns of activity (roughly speaking oscillations may go out of the phase-locked state for many short episodes or few long episodes). Recent developments in the time-series analysis allowed for the investigation of the temporal variations of phase-locking with a high temporal resolution [1]. Provided that there is some average level of phase-locking is present, one can follow oscillations from cycle to cycle and to observe if the phase difference is close to the preferred phase lag or not [1].

Recent study [2] of neural oscillations in basal ganglia in Parkinson's disease revealed the patterning of neural phase-locking: the synchronized state was interrupted by numerous but mostly short desynchronization states.

**Figure 1:**
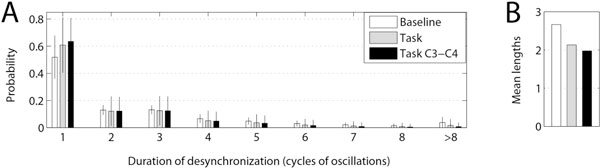
**(A) distribution of desynchronization durations and (B) mean desynchronization duration at the beta band for all EEG electrodes at rest (Baseline) and during motor task (Task) as well as for C3 and C4 electrodes (Task C3-C4)**.

Here we study neural oscillations recorded by EEG in alpha and beta frequency bands in a large sample of healthy human subjects at rest and during the execution of a simple motor task. While the phase-locking strength depends on many factors, dynamics of synchrony has a very specific temporal pattern: synchronous states are interrupted by frequent, but short desynchronization episodes. The probability for a desynchronization episode to occur decreased with its duration (see Figure 1). The modes and medians of distributions of desynchronization durations were always just one cycle of oscillations.

Similar temporal patterning of synchrony in different brain areas in different states may suggest that i) this type of patterning is a generic phenomenon in the brain, ii) it may have some functional advantages for oscillating neural networks receiving, processing, and transmitting information, iii) it may be grounded in some general properties of neuronal networks calling for the development of appropriate nonlinear dynamical theory.

To further investigate these conjectures we numerically studied a system of two and three coupled simple neuronal models (of Morris-Lecar type) and showed that fast kinetic of ionic conductances leads to the emergence of short desynchronized events when the coupling strength is below the full synchronization threshold. We further show that coupled neural oscillators exhibiting short desynchronizations require smaller values of synaptic connections between them of weaker common synaptic input to induce specified levels of synchrony strength than oscillators of the same frequency exhibiting more prolong desynchronizations.

The results may suggests that whenever a (partially) synchronous cell assembly must be formed to facilitate some function, short desynchronization dynamics may allow for efficient formation and break-up of such an assembly.
